# Invalidation of Microsomal Prostaglandin E Synthase-1 (mPGES-1) Reduces Diet-Induced Low-Grade Inflammation and Adiposity

**DOI:** 10.3389/fphys.2018.01358

**Published:** 2018-10-02

**Authors:** Clément Pierre, Florent Guillebaud, Coraline Airault, Nathalie Baril, Rym Barbouche, Etienne Save, Stéphanie Gaigé, Bruno Bariohay, Michel Dallaporta, Jean-Denis Troadec

**Affiliations:** ^1^Aix Marseille Université, CNRS, Laboratoire de Neurosciences Cognitives UMR 7291, Marseille, France; ^2^Biomeostasis CRO, La Penne-sur-Huveaune, France; ^3^CNRS, Fédération de Recherche 3C FR 3512, Aix-Marseille Université, Marseille, France

**Keywords:** obesity, high-fat diet, mPGES-1, prostaglandins, PGE2, adipose tissue

## Abstract

Chronic low-grade inflammation is known to be linked to obesity, and to occur in the early stages of the disease. This mechanism is complex and involves numerous organs, cells, and cytokines. In this context, inflammation of white adipose tissue seems to play a key role in the development of obesity. Because of its properties, prostaglandin E2 (PGE2), an emblematic inflammatory mediator, has been proposed as an actor linking inflammation and obesity. Indeed, PGE2 is involved in mechanisms that are dysregulated in obesity such as lipolysis and adipogenesis. Microsomal prostaglandin E synthase-1 (mPGES-1) is an enzyme, which specifically catalyzes the final step of PGE2 biosynthesis. Interestingly, mPGES-1 invalidation dramatically alters the production of PGE2 during inflammation. In the present work, we sought to determine whether mPGES-1 could contribute to inflammation associated with obesity. To this end, we analyzed the energy metabolism of mPGES-1 deficient mice (mPGES-1^-/-^) and littermate controls, fed with a high-fat diet. Our data showed that mPGES-1^-/-^ mice exhibited resistance to diet-induced obesity when compared to wild-type littermates. mPGES-1^-/-^ mice fed with a high-fat diet, showed a lower body weight gain and a reduced adiposity, which were accompanied by a decrease in adipose tissues inflammation. We also observed an increase in energy expenditures in mPGES-1^-/-^ mice fed with a high-fat diet without any changes in activity and browning process. Altogether, these data suggest that mPGES-1 inhibition may prevent diet-induced obesity.

## Introduction

The rapid increase in the worldwide prevalence of the metabolic syndrome, including obesity and diabetes, is a major public health problem for decades (World Health Organization, 2000). Faced with such an epidemic, the therapeutic options remain limited and inefficient, notably because of a lack of a complete understanding of the mechanisms underlying the obesity development (Bariohay et al., 2011). Among these, chronic low-grade inflammation seems to be of particular interest. Indeed, obesity is strongly associated with an increase in pro-inflammatory signals production, including many cytokines and lipid mediators. In the long term, these effectors become deleterious and maladaptive, by creating health impairments, such as dyslipidemia, cardiovascular diseases, and type-2 diabetes (Ferguson et al., 2013). Interestingly chronic low-grade inflammation appears to be instrumental in the development of obesity since inflammation-linked signals can be detected after the first days of high-fat feeding (Elgazar-Carmon et al., 2008; Thaler et al., 2012). In accordance, the blocking of certain signaling systems downstream of these cytokines is able to prevent the appearance of obesity conventionally observed in mice subjected to a high calorie diet (Hotamisligil, 2006; Thaler et al., 2012; Guillemot-Legris and Muccioli, 2017).

Prostaglandin E2 (PGE2) has been proposed to belong to the many factors contributing to the uncontrolled inflammation observed in obese individuals (González-Périz and Clària, 2010). PGE2 is a member of the eicosanoid family, which is derived from arachidonic acid and other polyunsaturated fatty acid and is ubiquitously expressed in mammals (Pecchi et al., 2009). The last step of PGE2 production is catalyzed by three different PGE synthases (PGES). The cytosolic PGES, and the microsomal PGES-2 are constitutively expressed, while the microsomal PGES-1 (mPGES-1) has been shown to catalyze the formation of PGE2 in response to inflammatory cytokines (Pecchi et al., 2009), and is preferentially coupled with cyclo-oxygenase 2 (COX-2). We have contributed with others to show that mPGES-1 is essential for behavioral changes observed during acute or subacute inflammation (Engblom et al., 2003; Pecchi et al., 2006, 2008, 2009). Interestingly, Hétu and Riendeau (2007) reported that mPGES-1 expression was downregulated in white adipose tissue (WAT) of obese mice. Emergent data indicates that PGE2 production is modified during obesity but the precise contribution of PGE2 in the development of obesity and associated complications is ambiguous. Indeed, PGE2 was shown to exert an anti-lipolysis effect in humans and mice (Richelsen and Pedersen, 1987; Ceddia et al., 2016) and thus proposed to facilitate adipose tissue lipid accumulation. Consistently, many works have considered the contribution of COX-2-derived PGs in obesity development, and evaluated the benefit of pharmacological or genetic COX-2 inhibition on diet-induced obesity (Hsieh et al., 2010; Chan et al., 2016; Rossi et al., 2018). COX-2 and PGE2 receptor-3 (EP3) inhibitors reversed obesity-induced adipose tissues inflammation and obesity-linked complications (Chan et al., 2016). Moreover, loss of PGs production in adipose tissue by deletion of adipocyte phospholipase (AdPLA) was shown to increase lipolysis, and AdPLA^-/-^ mice were shown to be resistant to diet-induced obesity (Jaworski et al., 2009). Otherwise, PGE2 was shown to exert an anti-adipogenic effect via its EP3 receptor (Xu et al., 2016). In accordance, EP3^-/-^ mice develop a more robust obese phenotype when fed with a high-fat diet (HFD; Sanchez-Alavez et al., 2007; Ceddia et al., 2016). Surprisingly, the role of mPGES-1 throughout the development of obesity remains poorly studied while mPGES-1 has been shown to specifically catalyze PGE2 formation in response to inflammatory cytokines (Pecchi et al., 2009).

In this context, the aim of this study was to decipher the role PGE2 in chronic low-grade inflammation associated with obesity development by specially targeting mPGES-1. To this end, we took advantage of mPGES-1 deficient mice (Trebino et al., 2003) fed with a HFD and examined the effects of mPGES-1 deletion on body weight (BW) gain, energy expenditures (EE), adiposity development and adipose tissues inflammation.

## Materials and Methods

### Impact of High-Fat Diet on mPGES-1 Expression

Experiments were performed on C57BL/6 male mice at 5 weeks of age (Charles River, France). Following receipt, animals were fed with standard diet (AO4, SAFE UAR, France) and water *ad libitum* for 1 week. Then, animals were given free access to either normal chow (NC, *n* = 10) or a HFD (energy content: 60% from fat ssniff^®^ EF acc. D12492 (I) mod diet, ssniff Spezialdiäten GmbH, Germany; *n* = 11) for 10 weeks.

### Impact of mPGES-1 Deletion on Obesity Development

Experiments were performed on adult male mice of the DBA/11ac J strain, with deletion of the PTGES-1 gene, which encodes mPGES-1 enzyme (Pfizer, Trebino et al., 2003). mPGES-1^-/-^ (KO) mice and wild-type (WT) mice were obtained from heterozygote mating. At 10–12 weeks of aged, mice were given free access to NC and water available *ad libitum* for 1 week (from Day 7 to Day 1). Then, seven WT and six KO mice were fed with NC, while eight WT and six KO mice were fed with a HFD for 14 weeks (from Day 1 to Day 96). BW and food intake (FI) were measured twice a week. Cumulative FI was evaluated by subtracting the weight of the remaining food at the end of a defined period of time, to the pre-weighted quantity of food delivered at the beginning of this period. Semi-fasting glycemia (4 h of fasting) was measured at 5, 10, and 14 weeks of feeding, with an Accu-Chek^®^ Performa glucometer (Roche diagnostics, Meylan, France). This short-term fasting procedure was performed to normalize the metabolic status of the mice before the glycemia assay. EE and total physical activity were measured at Day-2 (D2), D43, and D70 during three consecutive days by indirect calorimetry. Magnetic Resonance Imaging (MRI) acquisition was performed at D85.

### Cold Exposure Experiments

Body core temperature was measured using implantable telemetry devices [ETA-F10, Data Sciences International (DSI), St. Paul, MN, United States], and data acquisition was performed using Dataquest A.R.T. software (version 4.31; DSI). WT (*n* = 8) and KO (*n* = 7) mice fed with NC were anesthetized with isoflurane (2%) and telemetry devices were inserted in the abdominal cavity. Then, animals were individually housed, and allowed to recover for 10 days. Following this recovery period, animals were exposed to cold (8°C +/- 2°C) during three consecutive days. The body core temperature was continuously assessed at an interval of 30 s, every 5 min. At the end of cold exposure, animals were euthanized by cervical dislocation before immediate tissue collection.

### Organ Samples

Animals were fasted during 4 h, and then, anesthetized by intraperitoneal injection of ketamine (100 mg/kg; Imalgen 1000, Merial, France) and xylazine (10 mg/kg; Rompun, Bayer Santé, France), to be euthanized by cardiac puncture. Hypothalamus, dorsal brainstem [i.e., dorsal vagal complex (DVC)], liver, epididymal WAT (EWAT), retroperitoneal WAT (RWAT), interscapular brown adipose tissue (iBAT), and blood were immediately collected and frozen in liquid nitrogen and kept at -80°C for later analysis.

### Measurement of Energy Expenditures and Total Physical Activity

BW and FI were daily measured, before the onset of the light phase. Oxygen consumption (VO_2_), and carbon dioxide production (VCO_2_) were measured using the Oxylet Physiocage System (Panlab/Harvard apparatus, Cornellà, Spain) and the software suite METABOLISM (V2.2.01, Panlab). The respiratory exchange ratio (RER) was calculated as VCO_2_/VO_2_ and EE was calculated according to the formula EE [kcal/(day.kg)] = VO_2_ × 1.44 × [3.815 + (1.232 × RER)]. Mice were singled housed and habituated to the metabolic chambers for 24 h before data collection for 48 h. Calorimetric analyses were performed at three time points during the feeding period (a time line depicting the protocol used is given in **Figure 2A**. The mean values of VO2, VCO2, RER, and EE obtained during D1, D45, and D72 were compared to baseline data obtained for each animal at the beginning of the experiment (D-1) and were expressed in fold changes. Comparisons of EE were also conducted on light and dark phases. In addition, total physical activity was continuously measured.

### Quantitative PCR

Total RNA was extracted from frozen organ using TRI Reagent^®^ (Sigma-Aldrich) according to the manufacturer’s instructions. RT was realized using Moloney Murine Leukemia Virus Reverse Transcriptase in the presence of random hexamer primers (Promega). Gene expression analysis by real-time PCR was performed using the LightCycler^®^ 480 System (Roche Applied Science). The equivalent of 20 or 40 ng initial RNA was subjected to PCR amplification with a 10 μL final volume using specific 0.5 μM primers and SYBR green PCR Master Mix (Applied Biosystems). The generation of specific PCR products was confirmed by melting-curve analysis. U6 and glyceraldehyde-3-phosphate dehydrogenase gene (GAPDH) were used as internal reference gene.

### *In vivo* Magnetic Resonance Imaging Acquisition

Experiments were performed on a 70/16 pharmascan spectrometer (BRUKER Biospin, Ettlingen, Germany) equipped with a 7-Tesla magnet and 16-cm horizontal bore size. A linear birdcage coil with 38-mm inner diameter was used for signal transmission and reception. To perform *in vivo* anatomical magnetic resonance images at D85, mice were fasting overnight and then anesthetized using a mixture of air (2 L/min) and isoflurane, 3% for induction into a hermetic cage, and 2% for maintenance via a nose-cone of a head-holder device. Two sets of 22–26 coronal contiguous T1-weighted images (slice thickness = 1 mm) were acquired on the front and on the back part of the mice body with a turbo-RARE sequence (TE = 7.5 ms, TR from 850 to 1200 ms depending on the slices number, rare factor = 4, 1 average) using a 50 mm Field Of View and 256 × 256 matrix. A pressure probe monitored mice respiration. Analyses of adiposity were performed as previously described (Ke et al., 2015) with Image J Software (NIH, United States).

### Histological Analysis

EAT was immediately fixed with 4% paraformaldehyde in 0.1 M phosphate buffer at 4°C overnight and rinsed in 0.1 M phosphate buffer saline (pH 7.4). EAT were cryoprotected in 30% sucrose solution and frozen in OCT embedding matrix (Cell Path, United Kingdom). 8 μm coronal sections were prepared using a cryostat (Leica, CM3050). Images were acquired using a 10-fold lens with a DMX 1200 camera (Nikon) coupled to ACT-1 software. At least 150 adipocytes were counted for each mouse. Cell surfaces were obtained by dividing the number of cells by the surface of the observed area (0.075 mm^2^).

### SDS-PAGE and Western Blot Analysis

Adipose tissues were homogenized in RIPA buffer (50 mM Tris pH 8.0, 0.1% sodium dodecyl sulfate, 1% Triton X-100, 0.5% sodium deoxycholate, 150 mM NaCl, and 1 mM EDTA) supplemented with protease inhibitors cocktail (Sigma, France) then maintained under constant agitation in ice for 1 h. Extracts were centrifuged at 12,000 × *g* for 20 min at 4°C to remove tissue debris. Protein concentration was performed with BCA Protein Assay Kit (Novagen). Soluble protein extracts (30 μg) were separated by 15% SDS-polyacrylamide gel electrophoresis and transferred to nitrocellulose membranes (Amersham). Blots were blocked for 30 min at room temperature with 2% casein in PBS-T and incubated 2 h at room temperature with rabbit polyclonal antibody against mPGES-1 (18 kDa) at 1:1000 dilution (Oxford BioMedical Research, United States) and with mouse monoclonal antibody against GAPDH (36 kDa) at 1:10000 dilution (Proteintech, United States). Blots were then incubated for 1 h at room temperature with anti-rabbit peroxidase conjugated secondary antibodies at 1:1000 dilution (Dako) and with anti-mouse IgG (Fab specific)-peroxidase at 1:10000 dilution (Sigma) and visualized using the colorimetric system TMB-Blotting (Thermoscientific, France). Bands were quantified by densitometry using Image J software (NIH, United States).

### Analysis of Plasma Samples

Plasma was immediately separated by centrifugation (3000 × *g*, 15 min, 4°C). Plasma leptin levels were measured using ELISA (Eurobio, France) according to the manufacturer instructions.

### Statistical Analysis

All results are presented as mean ± SEM. Statistical analyses were performed using StatView (version 5.0.1.0, StatView Software) with repeated measures ANOVA followed by Bonferroni’s multiple comparisons for daily FI and BW gain. Significant difference was assessed by a one-way ANOVA followed by a *post hoc* Fisher’s test for comparison between four groups. Comparison between two groups was performed using unpaired 2-tailed Student’s *t*-test. Pearson correlation analysis was used to quantify relationships between variables of interest. *P*-values less than 0.05 were considered significant.

## Results

### mPGES-1 Expression Is Decreased in Mice Fed With a High-Fat Diet

**FIGURE 1:**
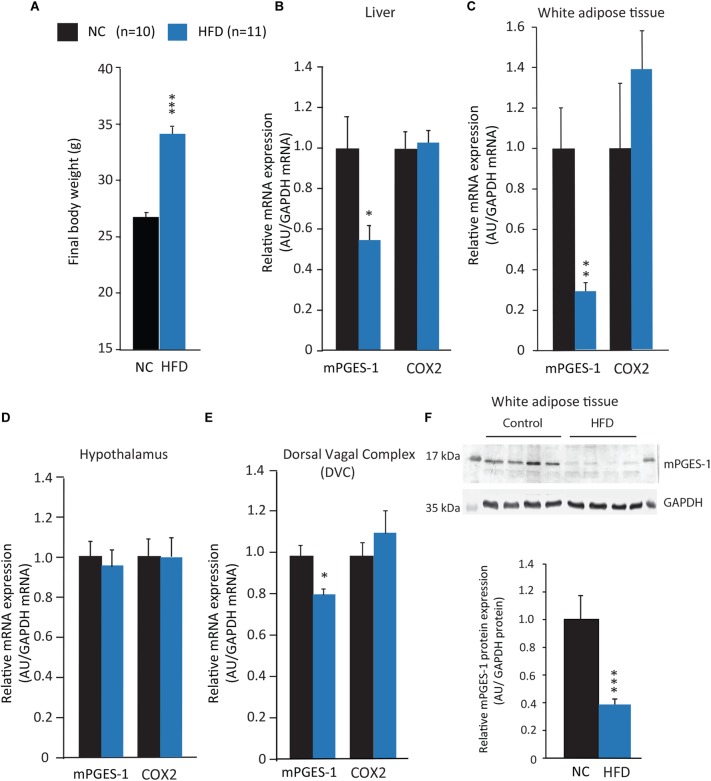
mPGES-1 expression is reduced in diet-induced obesity model. **(A)** Final BW (g) of C57BL/6 mice fed with either normal chow (NC) or a high-fat diet (HFD) for 10 weeks. **(B–E)** Real-time PCR analysis of mPGES-1 and COX-2 mRNA expression in the liver **(B)**, white adipose tissue **(C)**, hypothalamus **(D)** and brainstem **(E)** in NC- and HFD-fed mice. **(F)** Western blot analysis of mPGES-1 expression within the white adipose tissue of NC- and HFD-fed mice. First and last lanes: 17 kDa weight ladder. Data shown are mean ± SEM; ^∗^, significantly different from NC; ^∗^*p* < 0.05; ^∗∗^*p* < 0.01; and ^∗∗∗^*p* < 0.001.

To investigate the impact of obesity on mPGES-1 expression (**Figures 1A–F**), 5-week-old C57BL/6 male mice were fed with NC or a HFD for 10 weeks. As expected, BW of HFD-animals was increased by 26.4% when compared with NC-animals (**Figure 1A**). As shown in **Figures 1B,C**, mRNA expression of mPGES-1 was significantly decreased in liver and WAT, without modification of COX-2 mRNA expression. The decrease in mPGES-1 expression was confirmed at the protein level by western blot analysis in EWAT (**Figure 1F**). At the brain level, mRNA expression was evaluated within two central structures involved in the regulation of appetite, i.e., the hypothalamus and DVC. While any differences were observed in the hypothalamus (**Figure 1D**), a small but significant decrease in mPGES-1 mRNA expression was detected in the DVC (**Figure 1E**). These data confirmed and deciphered previous results showing a decreased mPGES-1 expression in adipose tissue of HFD-fed mice (Hétu and Riendeau, 2007). The specific mPGES-1 decreased expression, observed in key metabolic organs led us to characterize the sensitivity of mPGES-1^-/-^ mice to develop diet-induced obesity.

### mPGES-1^-/-^ Mice Are Resistant to High-Fat Diet Induced Obesity

**FIGURE 2:**
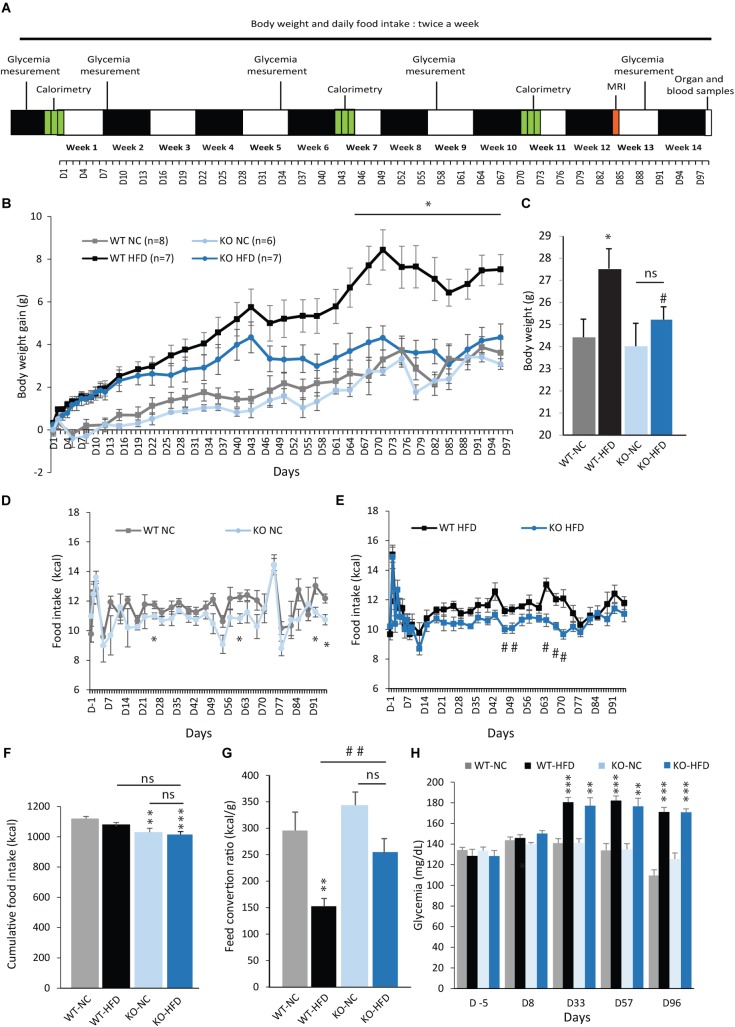
mPGES-1 KO mice are resistant to diet-induced weight gain. **(A)** Time line illustrating the measurements performed during the feeding period. **(B)** BW gain (g) and final BW **(C)** of WT type and mPGES-1^-/-^ mice fed either with normal chow (NC) or a high-fat diet (HFD) for 14 weeks. **(D,E)** Daily food intake (kcal) of WT type and mPGES-1^-/-^ mice fed either with NC or HFD. Cumulative food intake (**F**, kcal) and feed conversion ratio (**G**, kcal/g) of WT type and mPGES-1^-/-^ mice calculated at the end of 14 weeks. **(H)** Fasting glycemia (mg/dL) measured for all groups at different time points of the feeding period. Data shown are mean ± SEM; ^∗^, significantly different from WT-NC; ^#^, significant difference between WT-HFD and KO-HFD; ^∗^*p* < 0.05; ^∗∗^*p* < 0.01; ^∗∗∗^*p* < 0.001; same statistical parameters apply to ^#^ symbol; and ns, not significant.

To explore the impact of mPGES-1 deletion on obesity development, mPGES-1^-/-^ mice and control littermates were fed with either NC or a HFD for 14 weeks. In order to follow the evolution of the obese phenotype, we performed several measures of BW, FI, semi-fasted glycemia, and EE throughout the diet period. A MRI acquisition was also performed after 12 weeks of diet. A time line depicting the protocol used is given in **Figure 2A**. As shown in **Figures 2B,C**, no differences in BW gain were observed between WT and KO mice when fed with NC. As expected, WT-HFD mice presented a rapid increase in BW gain, which was significant from D2 to the end of diet period. Notably, the final BW of WT-HFD mice was significantly increased by 9% compared with WT-NC mice (**Figures 2B,C**). During the first week of diet, KO mice fed with a HFD exhibited similar BW gain than WT-HFD mice. But thereafter, their BW gain increased slower and became significantly lower than those of WT-HFD mice after 10 weeks (**Figures 2A,B**). Interestingly, there was no significant difference in final BW between KO-HFD mice and mice fed with NC, indicating a resistance to HFD-induced obesity. Despite a similar BW gain, the FI of KO-NC mice trended to be lower, or, was punctually significantly lower (D-26, -61, -92, and -96) than those of WT-NC mice (**Figure 2D**). Similar results were observed between WT-HFD and KO-HFD mice (**Figure 2E**). Consequently, the cumulative FI of KO-NC and KO-HFD mice was slightly but significantly lower compared with WT-NC mice. Nevertheless, there was no statistical difference in cumulative FI between WT-HFD and KO-HFD mice (**Figure 2F**). Therefore, the phenotype of KO-HFD cannot be entirely explained by a lower FI. However, the feed conversion ratio of KO-HFD mice was significantly higher than WT-HFD mice (**Figure 2G**) showing that mPGES-1^-/-^ mice are less efficient than their littermates, to convert energy from fat for growth. The resistance to diet-induced obesity observed in KO mice was not accompanied by an improvement of glycemia profile. Indeed, both WT-HFD and KO-HFD presented a similar increase in semi-fasted glycemia measured at three points of the feeding period, i.e., D-33, -57, and -96 (**Figure 2H**).

### Deletion of mPGES-1 Reduced HFD-Induced Adiposity

**FIGURE 3:**
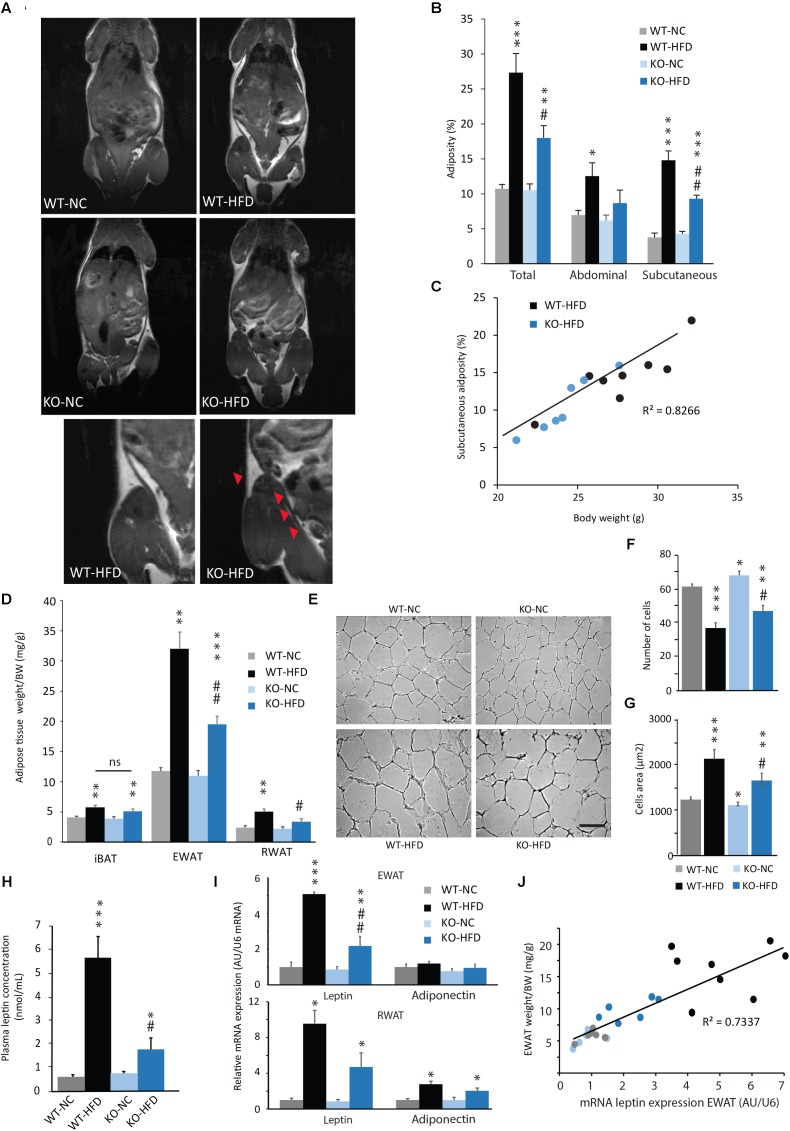
Reduced adiposity in HFD-fed mPGES-1 KO mice. **(A)** Coronal MRI analysis of WT and KO mice fed with normal chow (NC) or a high-fat diet (HFD) for 14 weeks. Slice of representative mice are shown. Detail images focus on reduced subcutaneous adiposity (arrowheads) in HFD-fed mPGES-1 KO mice compared with HFD-fed WT mice. **(B)** Quantification of total, visceral and subcutaneous adiposities (%) of 14-week-fed WT and KO mice. **(C)** Correlation analysis was performed on the body weight and subcutaneous adiposity of HFD-fed WT and KO mice (*R*^2^ = 0.82). **(D)** Weights (g/BW) of interscapular brown adipose tissue (iBAT), epididymal white adipose tissue (EWAT) and retroperitoneal white adipose tissue (RWAT), in WT and KO mice fed either with NC or a HFD for 14 weeks. **(E)** Representative photomicrographs of EWAT histology in WT and KO mice fed either with NC or a HFD. Quantification of adipocytes number per surface unit **(F)** and adipocytes area (**G**, μm^2^) in EWAT of WT and KO mice fed either with NC or HFD. **(H)** Dosage of plasmatic leptin (nmol/mL) in all experimental groups. **(I)** Real-time PCR analysis of leptin and adiponectin mRNA expression in EWAT and RWAT from WT and KO mice fed either with NC or HFD. **(J)** Correlation analysis was performed on the body weight and subcutaneous adiposity of WT and KO mice fed either with NC or a HFD (*R*^2^ = 0.73). Data shown are mean ± SEM. ^∗^, significantly different from WT-NC; ^#^, significant difference between WT-HFD and KO-HFD; ^∗^*p* < 0.05; ^∗∗^*p* < 0.01; ^∗∗∗^*p* < 0.001; same statistical parameters apply to ^#^ symbol; and ns, not significant. Scale bar: 100 μm.

To assess the development of adiposity in WT and KO mice fed with a HFD, we performed MRI acquisitions on animals after 12 weeks of diet. As expected, WT-HFD mice exhibited a significant increase in total, abdominal and subcutaneous adiposity, when compared with their control (**Figures 3A,B**). Interestingly, HFD-fed KO mice exhibited a significant lower total adiposity than WT-HFD mice (**Figures 3A,B**). This result was consistent with the reduced BW gain observed in KO mice during HFD feeding (**Figures 3B,C**). There was a strong linear association and correlation between the final BW and the total adiposity according to genotype and feeding status (*p* < 0.001, *R*^2^ = 0.82; **Figure 3C**). Abdominal adipose tissue is composed of different fat depots, including mesenteric, epididymal, perirenal, and ectopic adipose tissues. Thus, in complement of MRI analysis, EWAT and RWAT were dissected and weighted at the term of the feeding period (i.e., 14 weeks). We observed that the weight of EWAT and RWAT of KO-HFD mice was significantly lower than of WT-HFD mice (**Figure 3D**). In contrast, the mass of iBAT was increased by HFD feeding, without any difference between KO and WT mice (**Figure 3D**). As adipocyte hypertrophy is strongly correlated with adipocyte dysfunction (Goossens and Blaak, 2015), we then performed histological analysis on EWAT. As expected, a 14-week HFD induced a hypertrophy of WT and KO adipocytes, but this hypertrophy was significantly lower in KO mice when compared with WT mice (**Figures 3E–G**). In accordance with these results, the strong increases in both plasma leptin concentration and leptin mRNA expression observed in WT-HFD, were lessened in KO-HFD (**Figures 3H,I**). It should be noted that mRNA adiponectin expression was significantly increased in RWAT of HFD fed animals, but no difference was observed between WT and KO mice. Finally, the leptin mRNA expression was strongly and positively correlated with the weight of EWAT (**Figure 3J**) or RWAT (data not shown).

### HFD-Induced Inflammation of Adipose Tissues Was Reduced in mPGES-1^-/-^ Mice

**FIGURE 4:**
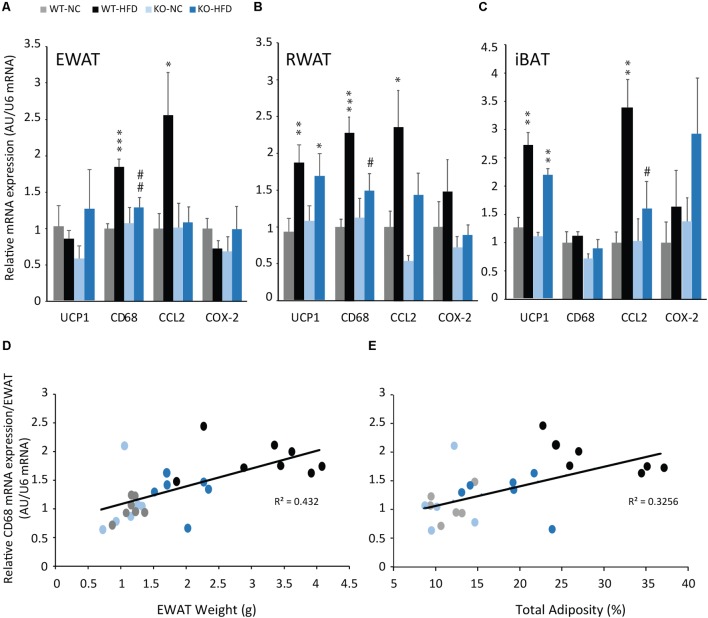
mPGES-1 invalidation reduces HFD-induced adipose tissues inflammation. Real-time PCR analysis of UCP1, CD68, CCL2 and COX-2 mRNA expression in EWAT **(A)**, RWAT **(B)**, and iBAT **(C)** of WT and KO fed either with normal chow (NC) or a high-fat diet (HFD). Correlation analysis were performed on the CD68 mRNA expression and EWAT weight (**D**, *R*^2^ = 0.432) and CD68 mRNA expression and total adiposity (**E**, *R*^2^ = 0.325). Data shown are mean ± SEM; ^∗^, significantly different from NC; ^#^, significant difference between WT-HFD and KO-HFD; ^∗^*p* < 0.05; ^∗∗^*p* < 0.01; ^∗∗∗^*p* < 0.001; and same statistical parameters apply to ^#^ symbol.

In order to examine whether decrease in adiposity observed in KO-HFD mice was associated with a decrease in inflammation, we analyzed the expression of pro-inflammatory markers by real-time PCR. As expected, the mRNA expression of pro-inflammatory macrophage infiltration markers (CD68, and CCL2) were significantly increased in iBAT, EWAT, and RWAT of WT mice fed with a HFD for 14 weeks, compared with WT-NC mice (**Figures 4A–C**). Interestingly, in KO-HFD mice, the increase in inflammation markers expression was totally blunted for CD68 (**Figures 4A,B**) or partially reversed for CCL2 in WAT (**Figures 4A,B**). Similarly, CCL2 mRNA expression was totally blunted in iBAT of KO-HFD (**Figure 4C**). The expression of inflammatory markers, i.e., CD68 mRNA expression was positively and significantly correlated with EWAT weight (**Figure 4D**, *R*^2^ = 0.423, *p* < 0.001) and total adiposity (**Figure 4E**, *R*^2^ = 0.33, *p* = 0.015).

### Energy Expenditures of mPGES-1^-/-^ Mice Are Improved During HFD Feeding

**FIGURE 5:**
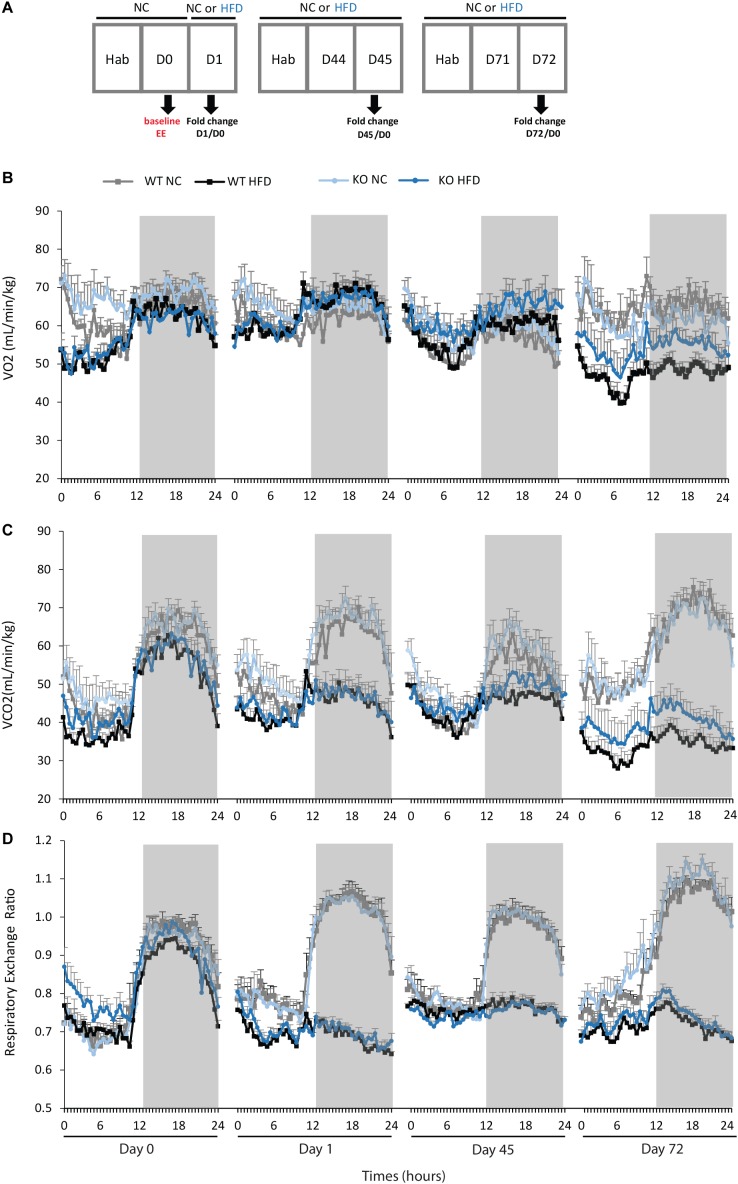
Calorimetry measurements of VO_2_, VCO_2_, and RER during HFD feeding. **(A)** Time line illustrating the protocol used for calorimetry measurements. WT and KO mice fed with either normal chow (NC) or a high-fat diet (HFD) were subjected to indirect calorimetry to assess VO_2_ (**B**, mL/min/kg), VCO_2_ (**C**, mL/min/kg) and RER **(D)**. Data shown are mean ± SEM.

**FIGURE 6:**
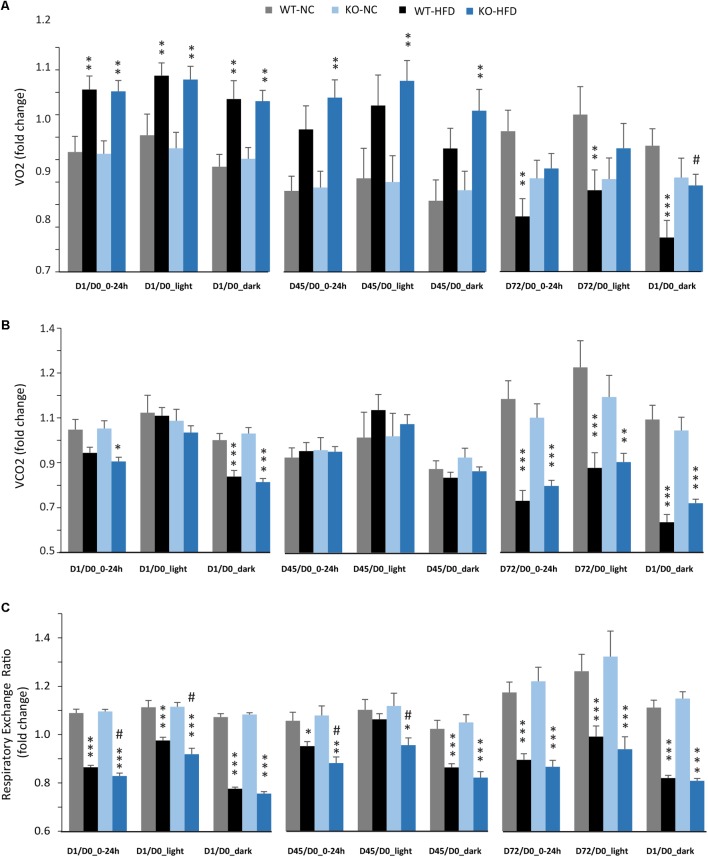
Modulation of VO_2_, VCO_2_, and RER during HFD feeding, impact of mPGES-1 deletion. Calorimetric analyses were performed at three time points during the feeding period. Each set of analysis comprised 3 days of monitoring; mean values were compared to baseline data obtained for each animal at the beginning of the experiment (D0) and expressed in fold changes for VO_2_ **(A)**, VCO_2_ **(B)**, and RER **(C)**. Data shown are mean ± SEM. ^∗^, significantly different from NC; ^#^, significant difference between WT-HFD and KO-HFD; ^∗^*p* < 0.05; ^∗∗^*p* < 0.01; ^∗∗∗^*p* < 0.001; and same statistical parameters apply to ^#^ symbol.

**FIGURE 7:**
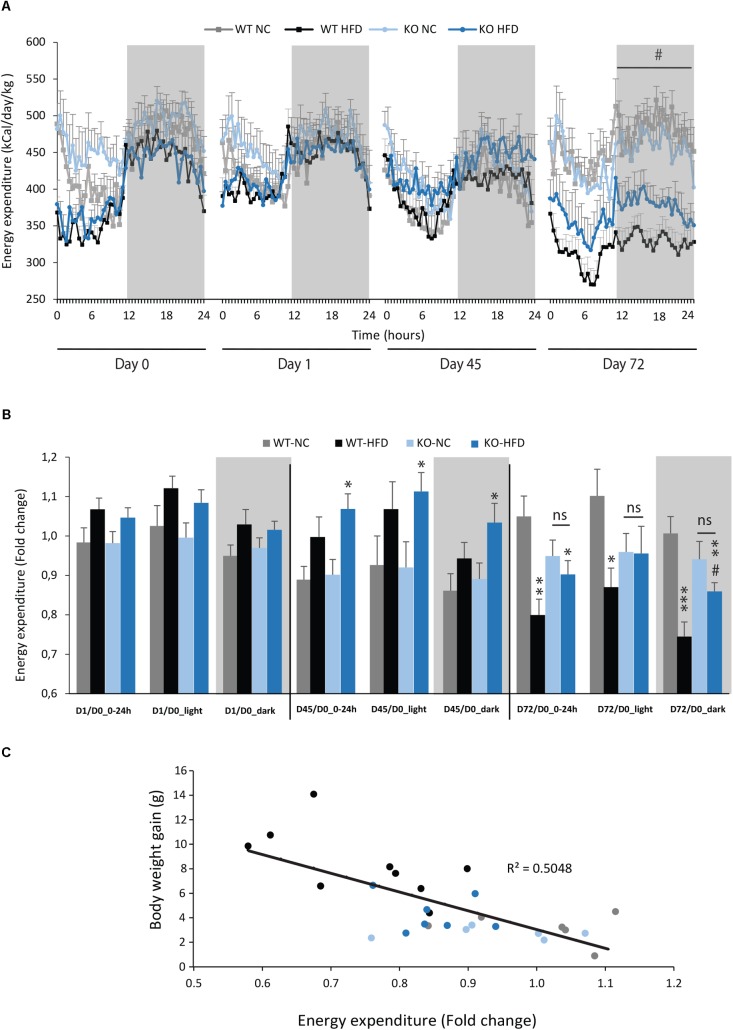
mPGES-1^-/-^ mice maintained their energy expenditure during HFD feeding. Energy expenditures (EE, kcal/day/kg) of WT and KO mice fed with either normal chow (NC) or a high-fat diet (HFD) were calculated from data obtained by indirect calorimetry. Data were acquired at three different time points (**A**; D1, D45, and D72). Mean total EE for light phase, dark phase, and the entire day were compared to baseline data obtained for each animal at the beginning of the experiment and expressed in fold changes **(B)**. A quantification of daytime and nighttime EE revealed significant differences between WT-HFD and KO-HFD mice. **(C)** Correlation analysis was performed on energy expenditures and body weight of WT and KO mice fed either with NC or HFD (*R*^2^ = 0.503). Data shown are mean ± SEM. ^∗^, significantly different from WT-NC; ^#^, significant difference between WT-HFD and KO-HFD; ^∗^*p* < 0.05; ^∗∗^*p* < 0.01; ^∗∗∗^*p* < 0.001; same statistical parameters apply to ^#^ symbol; and ns, not significant.

**FIGURE 8:**
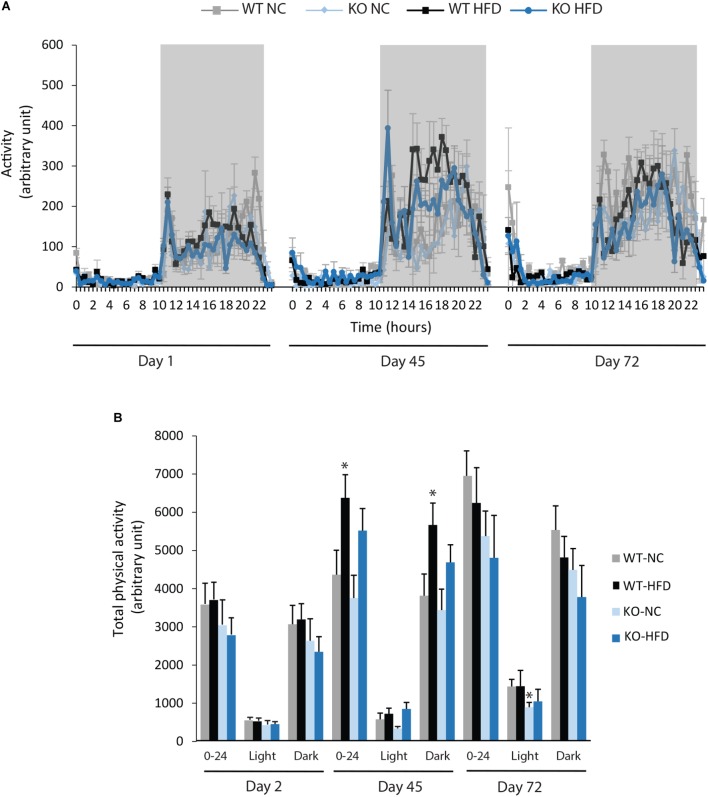
Deletion of mPGES-1 did not modify total physical activity. **(A)** Monitoring of physical activity for all groups at three different time points (D1, D45, and D72). **(B)** Quantification of total daily (0–24 h), daytime and nighttime physical activity. Data shown are mean ± SEM; ^∗^ significantly different from WT-NC; and ^∗^*p* < 0.05.

We then performed indirect calorimetry to assess VO2, VCO2, RER, and EE in WT and KO mice. A first measurement was carried out for all animals under standard diet. This measurement served as an internal reference for each animal and was referred as Day 0 (D0, **Figure 5A**). A longitudinal study was then carried out with measurements made at different times of the fat diet protocol (D-1, -45, and -72, **Figure 5A**). The choice of these time points was based on preliminary results obtained from a pilot experiment (data not shown). These points constitute crucial transitions during the feeding period, namely D1: beginning of HFD supply; D45: WT-HFD and KO-HFD began to show a difference in BW gain; D72: WT-HFD and KO-HFD showed a significant difference in BW gain. For each mouse, in addition to the raw data (**Figures 5B–D**), we made a ratio between the individual base values acquired at D0 and the different measurements obtained at D1, D45, and D72 (**Figure 6**). These results expressed in fold change illustrate the evolution of the different parameters during the fat diet. Switching animals from the control diet to a HFD induced an increase in VO_2_ and a decreased in VCO_2_ regardless of genotype considered (**Figures 5B,C**). These effects were observed from the first day of HFD feeding and were lessened at D45. On the other hand, at D72, both VO_2_ and VCO_2_ were reduced in WT-HFD compared with WT-NC, while only VCO2 was decreased in KO-HFD when compared with KO-NC (**Figures 5C**, **6B**). Consequently, VO2 measured during nighttime was significantly higher in HFD-KO than in HFD-WT (**Figure 6A**). In agreement with a HFD feeding and a preferential oxidation of lipid substrates, RER of HFD-mice no longer showed a clear day/night cycle and was comprised between 0.7 and 0.8 whatever the genotype and the time points considered (**Figures 5D**, **6C**). KO-HFD mice exhibited a slight and significant decrease in RER fold-change at D1 and D45 when compared with WT-HFD, but not at D72 (**Figure 6C**). EE analysis revealed a gradual and time-dependent decrease in EE of WT-HFD and KO-HFD-mice (**Figure 7A**). Indeed, EE of KO-HFD mice were increased at D45 compared to their respective controls. The same increase was observed for WT-HFD, but it was not significant. These increases in EE were mainly due to increases in total physical activity (**Figure 8**). Next, at D72, the EE of WT-HFD were significantly lesser than WT-NC (**Figures 7A,B**). Interestingly, this reduction in EE was less noticeable in KO-HFD mice that maintained a higher level of EE at D72 especially during the nighttime (**Figures 7A,B**). It should be noted that a strong and negative correlation was visible between BW gain and EE (**Figure 7C**, *R*^2^ = 0.505, *p* < 0.001). The EE difference observable between WT and KO mice fed with a HFD was not explained by changes in total physical activity (**Figure 8**).

### Invalidation of mPGES-1 Does Not Enhance Thermogenesis During Cold Exposure

**FIGURE 9:**
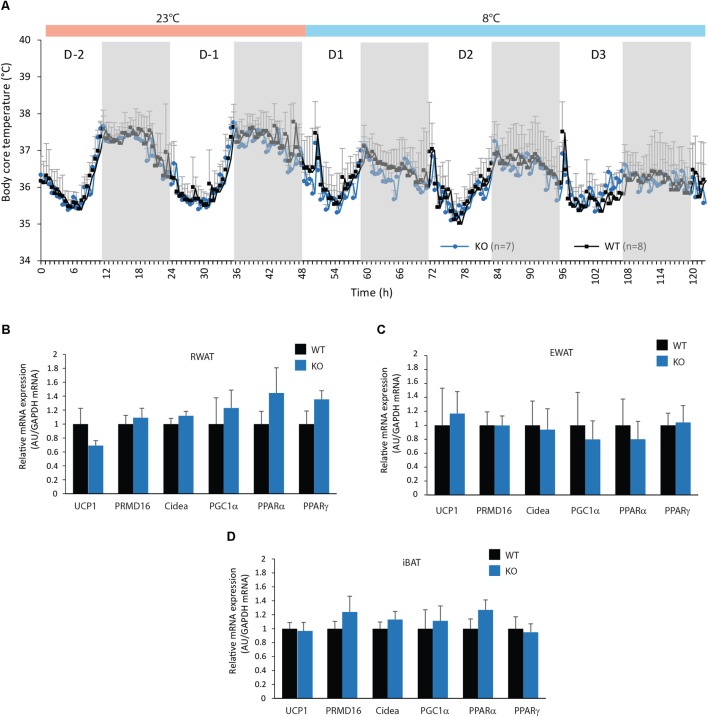
Figure mPGES-1 invalidation does not increase adipocytes browning. **(A)** Monitoring of body core temperature (°C) of WT and KO mice exposed at 23°C during 2 days and at 8°C the tree following days. **(B)** Real-time PCR analysis of UCP 1 and adipocytes differentiation related genes in the EWAT **(B)**, RWAT **(C)**, and iBAT **(D)** in WT and KO mice exposed to cold. Data shown are mean ± SEM.

Given the higher EE level observed in KO-HFD mice when compared to WT-HFD mice (**Figure 7**), we asked for a higher thermogenesis in KO mice revealed by HFD feeding. Moreover, during HFD feeding, numerous studies have reported that conversion of white adipocyte into brown adipocyte, a process known as browning, can impact EE (see for review Abdullahi and Jeschke, 2016). Chronic cold exposure was reported to exacerbate BAT thermogenesis and browning of WAT. Therefore, we analyzed the body core temperature and mRNA expression of browning markers in WAT and BAT of WT and KO mice after an exposition to cold (8°C, **Figure 9A**). No differences in body core temperature were observed between WT and KO mice, at room temperature (23°C) and under cold exposure (**Figure 9A**). In accordance, mRNA expression of both UCP1 and all browning markers tested were not significantly modified in RWAT, EWAT, and BAT of KO mice compared with WT mice (**Figures 9B–D**).

## Discussion

Chronic low-grade inflammation was reported to play a pivotal role in the pathogenesis of obesity (Hotamisligil, 2006). Thus, targeting inflammation appears as an attractive strategy to counter the burden of obesity and its associated comorbidities (Hardwick et al., 2013; Wang et al., 2016). Among potential targets, PGE2 appears of particular interest since previous works have reported their contribution to the uncontrolled inflammation observed in obese individuals (González-Périz and Clària, 2010). Accordingly, therapeutic strategies that specifically target enzymes catalyzing the production of PGE2 could be useful. Surprisingly, the mPGES-1 involvement in obesity-associated dysfunctions was not reported. The only clues that link mPGES-1 to obesity are rare and rely on a few studies reporting modulations of mPGES-1 expression during obesity. Moreover, these data are somewhat conflicted. Indeed, a liver mPGES-1 mRNA increase was reported in mice fed with a standard diet and water containing 30% of fructose for 8 weeks (Henkel et al., 2012). Otherwise, a down-regulation of mPGES-1 expression was observed in the EWAT of obese mice fed with a HFD (Hétu and Riendeau, 2007). Conversely, García-Alonso et al. (2016) described, in the human omental WAT of obese individuals, an increase in PGE2 expression due to an increase in COX-2 expression, without any changes in mPGES-1 expression. The discrepancy in reported results can be explained by the use of different species, tissues and energy sources. Consistently with the study of Hétu and Riendeau, we showed here a decrease in mPGES-1 expression in WAT of HFD-fed mice. Moreover, we observed that mPGES-1 mRNAs were also reduced in the liver and DVC of HFD-fed mice. As DVC is a dorsal brainstem structure involved in satiety control, it suggests an alteration of mechanism of FI control. Contrariwise, no modification of mPGES-1 mRNA expression was observed within the hypothalamus. This is quite surprising since the hypothalamus not only plays a key role in the regulation of energy balance, but was also reported to be subjected to inflammation during HFD feeding (Thaler et al., 2012). It is important to note that, the decreases in mPGES-1 expression reported here were not associated with a modification in COX-2 expression. The specific modulation of mPGES-1 during HFD-feeding questioned about a potential instrumental role of this enzyme in the mechanisms leading to obesity. Therefore, it appears that the use of mPGES-1^-/-^ mice would be of particular benefit, to shed light upon the possible role of mPGES-1 and PGE2 in obesity development. This animal model, characterized by a strong reduction of PGE2 production in inflammatory conditions (Trebino et al., 2003) was previously used to demonstrate the role of PGE2 and mPGES-1 in behavioral changes observed during acute inflammation (Elander et al., 2007; Siljehav et al., 2012).

The main result of the present study was the description of mPGES-1^-/-^ mice resistance to HFD-induced weight gain and development of adiposity. Indeed, after 14 weeks of fat diet, KO animals were not overweight compared to animals under low fat diet. In accordance, the adiposity of the KO mice was greatly reduced compared with the WT mice. Our results are to be compared to those of Jaworski et al. (2009) who reported that deletion of AdPLA2, which catalyzes the production of arachidonic acid, led to a lean phenotype in mice fed with a HFD for 15 weeks. However, AdPLA deletion doesn’t specifically downregulate PGE2 production because it is involved in numerous biosynthesis pathways. Conversely, others have described that EP3^-/-^ mice spontaneously developed an obese phenotype when fed with NC (Sanchez-Alavez et al., 2007; Xu et al., 2016). As EP3 is one the four different PGE2 receptors (EP1-4), it suggests that PGE2 exerts anti-lipolytic and anti-adipogenic action in WAT that through EP3. In our model of invalidation of PGE2 production, we find that mice exhibited a lean phenotype under HFD feeding, suggesting that EP2 and EP4 which had antagonistic effects to EP3, may also play a major role in the development of obesity. It should be noted that HFD-induced weight gain and development of adiposity observed in the DBA/11ac J mice strain used here, were lesser than those classically reported for other mice strains (Lin et al., 2000; Nasteska et al., 2014). The DBA/11ac J strain is not widely used in energetic metabolism studies and among the few data from the literature, it was reported that these mice were less susceptible to fat diet-induced BW gain (Wei et al., 2017). This is coherent with the moderate weight gain and the absence of hyperphagia observed here in HFD-fed DBA/11ac J mice. Despite this poor sensitivity to HFD-induced obesity, the deletion of mPGES-1 contributed to decrease adiposity.

We reported also a reduced macrophages infiltration in WAT of KO-HFD when compared with HFD-WT. Here, we showed that the increase in mRNA expression of pro-inflammatory macrophages (M1) infiltration markers was completely abolished in WAT of KO-HFD mice. This observation is critical since the balance impairment between M1, and anti-inflammatory macrophages (M2) was reported to trigger adipose tissue inflammation, and dysfunction throughout obesity (Wellen and Hotamisligil, 2003; Xu et al., 2003). This result is consistent with a previous work showing that PGE2 through their EP3 receptor, are involved in the release of pro-inflammatory cytokines within WAT (Chan et al., 2016). Conversely, PGE2 was also reported to shift macrophages polarization from M1 to M2 profile (Luan et al., 2015), mainly through EP4 (Yasui et al., 2015). These latest results are in contradiction with the reduced WAT inflammation described here within mPGES-1 KO mice, and we suggest that PGE2 pro-inflammatory action within the WAT is superior to its anti-inflammatory effect, during obesity development. Obesity is associated with an increased level of leptin prevailing in the expanding WAT. We showed here that deletion of mPGES-1 partially blunted both HFD-induced increase in plasmatic leptin levels, and WAT leptin mRNA expression. In accordance with these results, PGE2 is known to induce leptin secretion from rat adipocytes (Fain et al., 2000). But, this time again, the results of the literature are contradictory and EP3^-/-^ mice exhibited an increase in leptin circulating levels (Sanchez-Alavez et al., 2007). Altogether, these results suggest that PGE2-induce leptin release is not entirely controlled by EP3 receptor. Moreover, leptin is involved in both innate and adaptive immunity (La Cava and Matarese, 2004), suggesting a potential role of leptin in obesity-mediated inflammation (Tilg and Moschen, 2006). In accordance, leptin-deficient (ob/ob) and leptin receptor-deficient (db/db) mice have been shown less macrophagic infiltration and inflammatory gene expression in WAT, despite of a very significant increase in weight gain and adiposity (Xu et al., 2003). We cannot exclude that the reduction of adipose tissue inflammation observed in HFD-KO was partly due to the decreased leptin expression.

The weight gain curves showed that KO and WT animals evolved identically during the first 3 weeks of fat diets. Only after this delay, the weights of the KO animals begin to become significantly lower than WT mice after 8/10 weeks. From this moment, HFD-fed KO transgenic model began to have a lower BW gain than WT-HFD mice, and their final BW was comparable to mice fed with NC at the end of the diet period. This advocated for a gradual implementation of resistance to HFD-induced obesity in mPGES-1^-/-^ mice. In accordance, we observed that EE of HFD-mice varied with time of diet. First, EE tend or are significantly increased after 7 weeks of diet. One possible explanation is that increase in EE can contribute to counteract the increase in adiposity, and to regulate the excess of circulating fatty acids (Li et al., 2015). However, after 12 weeks of diet, EE of WT mice were importantly reduced during dark phase unlike KO mice, which conserved stables EE. The development of HFD-induced obesity consists of distinct phases of inflammation (Williams et al., 2014; Bai and Sun, 2015; Le Thuc et al., 2017). After a rapid induction of central and peripheral inflammatory markers (1–3 days), a second later phase appears after several weeks of HFD feeding (∼8 weeks). This second phase of inflammation in peripheral tissues, and especially in the WAT, causes a more intractable and persistent inflammation which is probably linked to lipid overload and lipotoxicity. In the light of the results we obtained, we proposed that the deletion of mPGES-1-derived PGE2 limits the inflammatory reactions that intervene during excessive lipid storage and growth of WAT. A similar profile was described for AdPLA2^-/-^ mice fed with a HFD that exhibited no difference in BW during the first 7–8 weeks of diet when compared with WT littermates (Jaworski et al., 2009). Interestingly, recent works have showed that anti-inflammatory treatments, especially those targeting macrophage polarization, lead to reduced BW gain (Kodama et al., 2015) and adiposity (Bashir et al., 2016; Toita et al., 2016; Tsai et al., 2018), in mice fed with a HFD. On the other hand, inflammation of adipose tissue is strictly linked to obesity development (Rogero and Calder (2018). Thus, we cannot exclude that the lower BW and adiposity observed in the HFD-fed KO mice, due to the maintenance of EE at a higher level, could explain the reduction of adipose tissue inflammation (Wensveen et al., 2015).

Here, we showed that the cumulative FI of KO-HFD mice was not different from those of WT-HFD mice, whatever the period of feeding considered. This was quite surprising since hypothalamus inflammation was reported to take place during obesity development and to contribute to overeating (Le Thuc et al., 2017). Our results suggest that mPGES-1-derived PGE2 does not contribute significantly to the inflammation at this level.

Non-shivering thermogenesis, a mechanism mainly driven by BAT, is well known to increase EE (Saito, 2013). This BAT function is associated with the strong expression of UCP1 within mitochondria, which disperses energy through heat production. Recent evidence has demonstrated that WAT can adopt a brown-like adipose tissue phenotype known as browning of WAT. This browning process is a potential new target for treating obesity and explains the resistance of different mice strains to diet-induced obesity (Wan et al., 2012; Schneider et al., 2016) by increasing non-shivering thermogenesis and enhancing EE (Yang et al., 2015; Choi et al., 2016). Some studies highlighted that PGE2 can activate the trans-differentiation of white adipocytes, into brown adipocytes, in both human and mice (García-Alonso et al., 2013; García-Alonso et al., 2016). As described in previous studies (So et al., 2011; García-Ruiz et al., 2015), we observed here that iBAT weight was increased in HFD mice but without any differences between WT and KO mice. The increase in iBAT mass can be promoted by ectopic fat deposition, leading to its inflammation and whitening (Gao et al., 2015). We also observed an increase in UCP1 mRNA expression in both iBAT and RWAT of mice fed with HFD. This could be a clue for an increased thermogenesis in response to lipid overload. However, this increase was similar in both genotypes. So, it seems unlikely that the increased EE observed in HFD-KO mice was associated with an increased browning of WAT, in the absence of mPGES-1-derived PGE2. To confirm this point, WT and KO mice were exposed to cold, which is a very effective protocol to boost browning and non-shivering thermogenesis. No difference between WT and KO mice was observed in body core temperature and browning markers expression. To conclude, we showed that the invalidation of mPGES-1 and the concomitant PGE2 decrease resulted in a less weight gain under HFD, and a moderate development of adiposity. We suggest that the absence of mPGES-1 prevents inflammation of WAT, inflammation known to contribute to the development of obesity and associated co-morbidities. We believe that a specific inhibition of mPGES-1 could constitute a therapeutic avenue against obesity that deserves to be tested.

## Ethics Statement

The protocols for the present study were authorized by the French Ministry, and approved by the Ethics Committee for animal experimentation of Marseille n°14, under the title “Obésité induite par un régime enrichi en graisse. Rôle de la prostaglandine E synthase microsomale (mPGES1)” with J-DT and BB as main investigators (reference number APAFIS#1955-2015092816241948, dated 10.27.2015).

## Author Contributions

CP, FG, CA, NB, RB, SG, and MD performed the experiments. CP, FG, CA, NB, RB, SG, ES, BB, MD, and J-DT designed the study and analyzed the data. CP and J-DT wrote the paper.

## Conflict of Interest Statement

The authors declare that the research was conducted in the absence of any commercial or financial relationships that could be construed as a potential conflict of interest.
